# Phytochemical Characterization and Pharmacological Properties of Lichen Extracts from Cetrarioid Clade by Multivariate Analysis and Molecular Docking

**DOI:** 10.1155/2022/5218248

**Published:** 2022-06-02

**Authors:** Isabel Ureña-Vacas, Elena González-Burgos, Simona De Vita, Padreep K. Divakar, Giuseppe Bifulco, M. Pilar Gómez-Serranillos

**Affiliations:** ^1^Department of Pharmacology, Pharmacognosy and Botany, Faculty of Pharmacy, Universidad Complutense de Madrid, Plaza Ramon y Cajal S/n, Ciudad Universitaria, Madrid 28040, Spain; ^2^Department of Pharmacy, University of Salerno, Via Giovanni Paolo II 134, Fisciano (SA) 84084, Italy

## Abstract

**Introduction:**

Lichens, due to the presence of own secondary metabolites such as depsidones and depsides, became a promising source of health-promoting organisms with pharmacological activities. However, lichens and their active compounds have been much less studied. Therefore, the present study aims to evaluate for the first time the antioxidant capacity and enzyme inhibitory activities of 14 lichen extracts belonging to cetrarioid clade in order to identify new natural products with potential pharmacological activity.

**Materials and Methods:**

In this study, an integrated strategy was applied combining multivariate statistical analysis (principal component analysis and hierarchical cluster analysis), phytochemical identification, activity evaluation (*in vitro* battery of antioxidant assays FRAP, DPPH, and ORAC), and enzyme inhibitory activity against acetylcholinesterase (AChE) and butyrylcholinesterase (BuChE) and molecular profiling with *in silico* docking studies of the most promising secondary metabolites*. Results*. Among fourteen lichen samples, *Dactylina arctica* stands out for its higher antioxidant capacities, followed by *Nephromopsis stracheyi, Tuckermannopsis americana*, *Vulpicida pinastri*, and *Asahinea scholanderi*. Moreover, *Asahinea scholanderi* and *Cetraria cucullata* extracts were the best inhibitors of AChE and BuChE. The major secondary metabolites identified by HPLC were alectoronic acid and *α*-collatolic acid for *Asahinea scholanderi* and usnic acid and protolichesterinic acid for *Cetraria cucullata*. Molecular docking studies revealed that alectoronic acid exhibited the strongest binding affinity with both AChE and BuChE with and without water molecules.

**Conclusions:**

Our results concluded that these species could be effective in the treatment of neurodegenerative diseases, being mandatory further investigation in cell culture and *in vivo* models.

## 1. Introduction

Lichens are a symbiotic association between a mycobiont (fungus) and a photosynthetic organism (algae and/or cyanobacteria). The use of lichens in traditional medicine has been fundamental to different cultures over the centuries. From classical traditional medicine systems (i.e., Ayurveda, Siddha, Unani, and Traditional Chinese Medicine (TCM)) to contemporary ethnic groups, lichens have been used for diverse medicinal purposes such as treating wounds, skin infections, respiratory, digestive, and gynecological diseases [1]. The estimated number of lichen species worldwide is around 28,000, being Parmeliaceae family the largest one of lichenized fungi (80 genera, 2,800 species). Among the five main clades (parmelioid, cetrarioid, usneoid, alectorioid, and hypogymnioid), cetrarioid clade stands out for the number of described genera (17 genera), second only to the parmelioid clade, which is the largest one (27 genera) [2].

Through traditional knowledge, it is known that some cetrarioid lichens have been used for different disorders via oral or topical administration. Hence, *Cetraria islandica* (L.) Ach. has been used for congestion, tuberculosis, asthma, inflammation, and high blood pressure [3, 4], Flavocetraria *cucullata* (Bellardi) Kärnefelt and Thell. for its antiasthmatic properties [4], *Nephromopsis nivalis* (L.) Divakar, A. Crespo and Lumbsch and *Vulpicida juniperus* (L.) J.-E. Mattsson and MJ Lai for its role as antibiotics [1], and *Vulpicida pinastri* (Scop.) J.-E. Mattsson and M. J. Lai for pulmonary tuberculosis, wounds, skin infections, cancer, and spasms [5].

In recent years, the scientific interest on the health-promoting benefits of lichens has grown as these organisms have shown interesting and promising activities including cytotoxic, antimicrobial, antioxidant, and anti-inflammatory [6–9]. These activities are attributed especially to the presence of own secondary metabolites such as depsidones and depsides [9]. However, studies focusing on therapeutic and protective strategy based on the antioxidant ability of lichens are very limited [10].

Altered cellular redox homeostasis due to an excessive reactive oxygen species (ROS) production and an impaired antioxidant system leads to oxidative damage of lipids, proteins, and DNA and even, cell death. Oxidative stress has been implicated in the pathogenesis of many diseases such as Alzheimer's disease [11]. Antioxidant compounds are good to prevent or delay oxidative stress-mediated toxicity through different mechanisms including accept or donate electrons to neutralize free radicals, upregulate the endogenous antioxidant system, and act as metal chelators [12, 13]. There is a growing interest in pharmaceutical, chemical, and food industries for natural antioxidants which is attributed to the tendency of society toward natural products and to the evidence of toxicity by synthetic antioxidants [14–16].

In addition to oxidative stress signaling in Alzheimer's disease, this major neurodegenerative disease has been associated with a deficiency in acetylcholine in brain. The enzymes acetylcholinesterase (AChE) and butyrylcholinesterase (BuChE) turn acetylcholine into the inactive metabolites, choline, and acetate. Therefore, inhibitors of cholinesterase enzymes are key in the prevention of Alzheimer's disease progression [17].

Since the pharmacological activity of lichens from cetrarioid clade has been scarcely studied, the purposes of the present work were (1) to evaluate the antioxidant activity and total phenol content of 14 lichen extracts, (2) to apply a multivariate analysis using principal component analysis (PCA) and hierarchical cluster analysis (HCA) to select which methanol lichen extracts have the best antioxidant activity, and (3) to evaluate AChE and BuChE enzyme inhibitory activities of all these 14 lichen extracts and assess molecular docking studies with the major secondary metabolites identified in the two most promising potential inhibitors lichen species for Alzheimer's disease.

## 2. Materials and Methods

### 2.1. Reagents

All reagents were acquired from Sigma-Aldrich (St. Louis, MO, USA). HPLC-grade methanol, ferrous sulfate (FeSO_4_), and dimethyl sulfoxide (DMSO) were purchased from Panreac (Barcelona, Spain).

### 2.2. Lichen Collection and Extract Preparation

**Figure 1:**
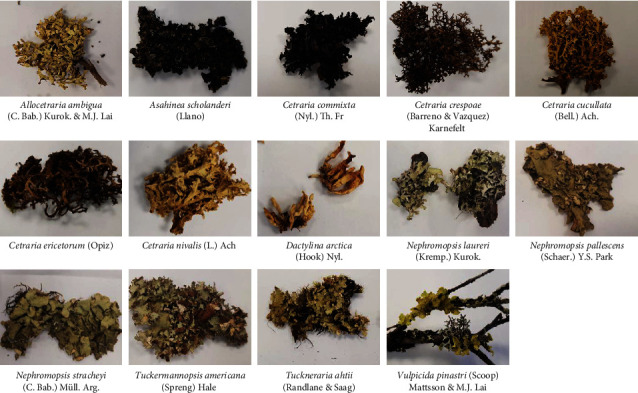
Thallus of the studied lichens of cetrarioid clade.

Lichen samples (Table 1, Figure 1) were collected in different countries and continents (America, Asia, and Europe). These samples were preserved in the Herbarium of the Faculty of Pharmacy (MAF), University Complutense of Madrid. Expert lichenologists (Dr. P.K. Divakar and Prof. A. Crespo) authenticated these species.

Lichen thallus (50 mg) was extracted successively (shaken 20 s, every 15 min) with pure methanol for HPLC (2 ml) during 2 h. After overnight maceration, extracts were filtered (0.45 *μ*m pore) and concentrated by evaporation at room temperature. Finally, dry extracts were stored until its use.

### 2.3. Total Phenolic Content

Total phenolic content was quantified by visible-light spectrophotometry using Folin–Ciocalteu colorimetric method [18]. Lichen extracts (1 mg/ml, 10 *µ*L) were mixed with Folin–Ciocalteu reagent (200 *µ*L). After 5-minute incubation, 7% Na_2_CO_3_ dissolution (90 *µ*L) was added. Gallic acid was used as standard for calibration curve. Absorbance was measured at 595 nm on a SPECTROstar Nano Microplate Reader (BMG Labtech, Ortenberg, Germany). Results were expressed as *µ*g of gallic acid equivalent per mg of extract.

### 2.4. In Vitro Antioxidant Assays

#### 2.4.1. Ferric-Reducing Antioxidant Power (FRAP) Assay

FRAP assay is based on the ability of antioxidants to reduce ferric ion into ferrous ion [19]. Lichen extracts (1 mg/ml in methanol, 110 *µ*L) were mixed with FRAP reagent [acetate buffer solution (pH 3.6), 2,4,6-Tris (2-pyridyl-s-triazine (TPTZ) (10 mM) in HCl (40 mM), and FeCl_3_ 6H_2_O (20 mM)]. A calibration curve of FeSO_4_ H_2_O was used as standard. After 30 min of incubation, spectrophotometric measurements at 595 nm were performed on a SPECTROstar Nano Microplate Reader. Results were expressed as *μ*mol of Fe^2+^ eq/g sample.

#### 2.4.2. 1,1-Diphenyl-2-Picrylhydrazyl (DPPH) Assay

Free radical-scavenging capacity was determined according to DPPH method [20]. This method is based on the ability of antioxidants to neutralize DPPH radical. Trolox was used as standard for calibration curve. Different concentrations of lichen extracts (from 100 to 900 mg/ml, stock solution 1 mg/ml in methanol) and DPPH solution (550 *µ*M, 20 *µ*L) were mixed and incubated at 37°C for 30 minutes. Absorbance was measured at 517 nm using a SPECTROstar Nano Microplate Reader. Inhibition of DPPH radical was expressed as IC_50_ values (*μ*g/mL).

#### 2.4.3. Oxygen Radical Absorbance Capacity (ORAC) Method

The ORAC assay was performed as previously described by Dávalos et al. (2004) [21]. Different concentrations of lichen samples from 10 to 500 *µ*g/ml (stock solution 1 mg/ml in methanol) were prepared in phosphate buffer solution (75 mM, pH 7.4) and incubated with sodium fluorescein (70 nM) for 10 minutes. Finally, 2,2'-azobis (2-amidinopropane)-dihydrochloride (APPH) was added to all samples. Fluorescence was recorded at 37°C for 98 min at 485 nm excitation wavelength and at 520 nm emission wavelength in a microplate reader (FLUOstar OPTIMA, BMG Labtech, Ortenberg, Germany). Results were expressed as *μ*mol TE/mg dry extract.

### 2.5. Enzyme Inhibitory Activities: Acetylcholinesterase (AChE) and Butyrylcholinesterase (BuChE)

The enzyme inhibitory activities AChE and BuChE were determined using Ellman method (1961) [22]. Lichen extracts (10 mg/ml in DMSO) were mixed at different concentrations (in PBS) with 5,5′-dithiobis (2-nitrobenzoic acid) (DTNB) (1.2 mM final concentration), AChE/BuChE (0.0008 U/well final concentration), Tris−HCl buffer (50 mM, pH 8.0), and bovine serum albumin (BSA) [(0.1% (w/v)]. After 5-min preincubation at room temperature, substrates acetylcholine iodide for AChE and butyrylthiocholine for BuChE (3 mM final concentration) were added. Absorbance was measured at 412 nm for 3 min in a SPECTROstar Nano Microplate Reader.

### 2.6. High-Performance Liquid Chromatography (HPLC)

Dry lichen extracts were dissolved in methanol (250 *µ*g/ml concentration). Samples were filtered twice and injected (20 *µ*L) in an HPLC instrumentation. The solvents were prefiltered under vacuum through 0.45 *μ*m pore size filters. The analysis was carried out using an Agilent 1260 instrument (Agilent Technologies, CA, USA), with a photodiode array detector (190–800 nm). The HPLC separations were performed on a reversed-phase Mediterranean Sea18 column at 40°C (150 mm × 4.6 mm, 3 *µ*m particle size; Teknokroma, Barcelona, Spain) using 1% orthophosphoric acid in milli-Q water (A) and supergradient HPLC-grade methanol (B) as solvents. The gradient elution with a flow rate of 0.6 ml/min started from 70% A to 30% after 15 min and down to 10% A after 45 min; initial conditions were reached after 60 minutes. The UV-Vis spectra and absorption maxima of sample components were recorded at 190–400 nm, and the peaks were detected at 254 nm. The analyses were run in duplicates. Agilent ChemStation was used to process chromatographic data [23, 24].

Main peaks were identified by comparing their retention times as well as the published spectroscopic data of scientific literature. The following standard compounds were used, and their *T*_*R*_ and *λ*_max_ were analyzed. Usnic acid was purchased from Sigma-Aldrich® (*T*_R_ = 32.39 min, *λ*_max_ = 232/282 nm). In addition, the extract of *Parmotrema nilgherrense* (Nyl.) Hale which contains alectoronic acid (*T*_*R*_ = 29.28 min, *λ*_max_ = 214/254/316 nm) and *α*-collatolic acid (*T*_R_ = 32.96 min, *λ*_max_ = 214–216/256/316 nm) and the extract of *Cetraria ericetorum* Opiz, which contains protolichesterinic acid (*T*_R_ = 41.2 min, *λ*_max_ = 230 nm), were used.

### 2.7. Docking Experiments

The crystal structures of human AChE bound to selective inhibitor donepezil (PDB: 6O4W [25]) and human BuChE bound to the inhibitor N-((1-(2,3-dihydro-1H-inden-2-yl) piperidin-3-yl) methyl)-N-(2-(dimethylamino) ethyl)-2 naphthamide (PDB: 5NN0 [26]) were considered for this study in a hydrated and a dehydrated state. The protein structures were prepared using Protein Preparation Wizard tool available in Schrödinger Suite (Schrödinger Suite 2017-1: Protein Preparation Wizard, Schrödinger, LLC, New York, NY, 2017) [27]. The chemical structures of acetylcholine (Ach) and the tested lichen secondary metabolites were built using Maestro Build Panel (Schrödinger Release 2017-1: Maestro, Schrödinger, LLC, New York, NY, 2017) and processed with LigPrep (Schrödinger Release 2017-1: LigPrep, Schrödinger, LLC, New York, NY, 2017).

The co-crystallized ligands and Ach were used as benchmarks for molecular docking experiments and to evaluate influence of water molecules inside binding cavity. Both inhibitors and Ach were re-docked against AChE and BuChE in the presence and in the absence of water molecules [25, 26]. The molecular docking experiments were carried out using Glide [28–30] in the extra precision mode (XP) software. The necessary grids were built assuming the center-of-mass of the ligand as the center of docking grid and expanding the box at 10 Å along *x-*, *y-*, and *z*-axis. The expanded sampling mode was used, and 10,000 ligand poses were kept for the initial phase, followed by a selection of 800 poses for energy minimization. In this step, 30 final poses were saved for each ligand, with a scaling factor of 0.8 related to van der Waals radii with a partial charge cutoff of 0.15.

### 2.8. Statistical Analysis

All assays were measured in triplicate, and data were expressed as mean ± standard deviation (SD). Statistical analysis was performed by Sigma Plot 11.0 using analysis of variance (ANOVA) and Tukey's post hoc test (5% significance level). Moreover, linear regression analysis and Pearson correlation coefficients were determined to correlate total phenolic content and antioxidant capacity.

Furthermore, a multivariate statistical analysis using principal component analysis (PCA) and hierarchical cluster analysis (HCA) was done using IBM SPSS statistics version 25. PCA is used to reduce the dimension of the data and to highlight the similarities and sort out the outliers. Due to the differences between the units of variables, we performed a PCA based on correlation matrix, in order to scale data and eliminate the influence of variances. PCA analysis model was carried out with a fixed number of factor (2) and choosing 25 maximum iterations for convergence. Unrotated factor solution and the scree plot were displayed. PCA score plot and loading plot were described. Hierarchical cluster analysis (HCA) was performed using the centroid method as clustering algorithm and the square Euclidean distance as distance measure. Variables were autoscaled (transformation into *z*-scores). Levene's test was carried out to check for homogeneity of variance. ANOVA tests were used to identify noted differences among the clusters. Lichen extracts have been grouped based on the antioxidant and phenolic content similarity. MVCA methods have been validated using leave-n-out cross-validation. Component's accountability and cos^2^ values were also calculated to validate the quality of representation by PCA model.

## 3. Results and Discussion

ROS overproduction is related to neurodegenerative disease progress via oxidative damage and mitochondria interaction [13]. Brain is especially susceptible to ROS damage because of its deficient level in antioxidant defenses, high oxygen consumption, presence of auto-oxidized neurotransmitter and redox active transition metals, and high content of polyunsaturated fatty acids in neuron membranes [12]. Since different ROS types are involved in neurodegenerative disease pathophysiology, the use of an exogenous combination of antioxidants is the most current and promising research strategy to deal with ROS injury [13]. Lichen extracts contain different compounds, most of them are exclusive, and they have polyphenolic structure with reported antioxidant properties. Parmeliaceae family is the largest one of lichenized fungi and, by number, highlights within it cetrarioid clade. The pharmacological research in cetrarioid clade is very limited. Therefore, the antioxidant activity of fourteen lichen extracts from cetrarioid clade was evaluated using different antioxidant assays.

Initially, extraction yields (dry extract weight/lichen thallus weight ^*∗*^ 100) were calculated for each methanol lichen extracts as shown in Table 2. The highest yields were found for *Dactylina arctica* (11.3%), *Asahinea scholanderi* (10.3%), and *Cetraria commixta* (10.3%). On the other hand, the lowest yield values were for *Cetraria nivalis* (5.6%) and *Cetraria crespoae* (6.2%).

Total phenolic content (TPC), using Folin–Ciocalteu method, was evaluated for all methanol lichen extracts. As shown in Table 2, the amount of total phenolics ranged from 39.3 *μ*g GA/mg for *Cetraria cucullata* to 113.5 *μ*g GA/mg for *Dactylina arctica*. The lichen species *Nephromopsis stracheyi* (84.2 *μ*g GA/mg) and *Asahinea scholanderi* (83.1 *μ*g GA/mg) also showed high total phenolics values. On the other hand, *Cetraria ericetorum* (41.7 *μ*g GA/mg), *Cetraria nivalis* (44.7 *μ*g GA/mg), *Cetraria commixta* (44.9 *μ*g GA/mg), and *Nephromopsis laureri* (45.1 *μ*g GA/mg) had low levels of phenolics. Similar results have been observed for other lichen species of cetrarioid clade such as *Cetraria islandica* and *Vulpicida canadiensis* which showed TPC values of 57.3 and 34.9 *μ*g GA/mg, respectively [10]. Moreover, total phenolic content has been also previously evaluated for lichen species of other clades such as parmelioid clade. For these lichens, total phenolic content ranges from 171 *μ*g GA/mg for *Parmotrema tiliacea* to 20 *μ*g GA/mg for *Parmotrema acetabulum* [31].

The antioxidant activity of lichen extracts was measured using three different methods: ferric-reducing antioxidant power (FRAP assay), 1,1-diphenyl-2-picrylhydrazyl (DPPH) assay, and oxygen radical absorbance capacity (ORAC) method (Table 2). Since there are multiple ROS inside living systems and *in vitro* methods for evaluation of antioxidant activity have different mechanisms of action, it is recommended to use diverse antioxidant test models [16, 32]. Hence, DPPH and FRAP methods evaluate the capacity of antioxidant molecules to transfer an electron to reduce radicals, metals, or/and carbonyls (single-electron transfer, SET), whereas ORAC assay measures the ability of antioxidant molecules to scavenge free radicals by proton donation (hydrogen atom transfer, HAT) [16]. DPPH radical-scavenging activity varied from 283.7 *μ*g/mL for *Vulpicida pinastri* to 2293.7 *μ*g/mL for *Cetraria crespoae.* Total antioxidant activity assayed by FRAP test ranged from 7.3 *μ*mol of Fe^2+^ eq/g sample for *Cetraria ericetorum* to 29.6 *μ*mol of Fe^2+^ eq/g sample for *Dactylina arctica*. Finally, the highest value for the ORAC test was obtained for *Dactylina arctica* (8.2 *μ*mol TE/mg dry extract) and the lowest ORAC value was for *Nephromopsis pallescens* (0.4 *μ*mol TE/mg dry extract). Previous studies have also demonstrated antioxidant properties for other cetrarioid species using DPPH and ORAC techniques. Hence, *Cetraria islandica,* which is the most studied species of the clade, showed DPPH values which varied from 678.38 *µ*g/mL to 1183 *µ*g/mL depending on the study [10, 33]. Moreover, *Vulpicida canadiensis* and *Vulpicida pinastri* exhibited significant DPPH results with IC_50_ values of 99 and 75 *µ*g/mL, respectively [10, 34]. Furthermore, regarding ORAC assays, *Cetraria islandica* has shown an ORAC value of 3.06 *μ*mol TE/mg dry extract and *Vulpicida canadiensis* of 0.77 *μ*mol TE/mg dry extract [10].

Beyond this clade, other clades of Parmeliaceae family have been investigated for its antioxidant activity. Hence, Sieteiglesias et al. evaluated a total of fifteen methanol lichen extracts from Parmelioid clade. The species with the highest antioxidant potential were *Hypotrachyna formosana* (DPPH value of 49.5 5 *μ*g/mL) and *Parmotrema perlatum* (FRAP value of 24.89 *μ*mol of Fe^2+^ eq/g sample and ORAC value of 22.1 *μ*mol TE/mg dry extract) [31]. Moreover, another study with Parmeliaceae lichens revealed that *Flavoparmelia euplecta* (ORAC value of 3.30 *μ*mol TE/mg dry), *Hypotrachyna cirrhata* (FRAP value of 316 *μ*mol Fe^2 +^ eq/g sample), and *Myelochroa irrugans* (DPPH value of IC_50_ 384 *μ*g/ml) were the most promising antioxidant lichen extracts [35].

Next, the extract antioxidant potency (EAP) index which is calculated as sample score/best score *x* 100 was determined to rank the antioxidant potency of each methanol lichen extract. The highest EAP index was for *Dactylina arctica* (93.5) followed by *Nephromopsis stracheyi* (66.8), *Vulpicida pinastri* (61.8), and *Tuckermannopsis americana* (59.6) (Table 2).

**Figure 2:**
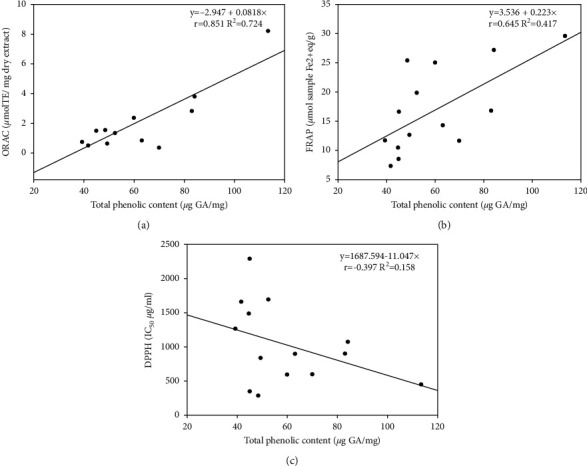
Linear correlation between total phenolic content and antioxidant capacity of lichens belonging to cetrarioid clade measured by (a) ORAC assay, (b) FRAP assay, and (c) DPPH assay.

To understand the bivariate correlation between phenolic content and each antioxidant method, the linear correlation coefficients (*r*) and the coefficient of determination (*R*^2^) were calculated. The study showed a significant and high correlation (*p* < 0.01) between phenolic content and ORAC assay (*r* = 0.851; *R*^2^ = 72.42%) and a significant and moderate correlation (*p* < 0.05) between phenolic content and FRAP method (*r* = 0.645; *R*^2^ = 41.60%). The lowest one was between DPPH and TPC (*r* = −0.397, *p* > 0.05) (Figure 2). Low correlation between total phenolic content and DPPH assay may be related to antagonistic or synergistic reactions between phenol compounds and other phytochemicals found in lichen extracts [36]. Flavonoids, tannins, and proanthocyanins could contribute to its antioxidant capacity [37].

**Figure 3:**
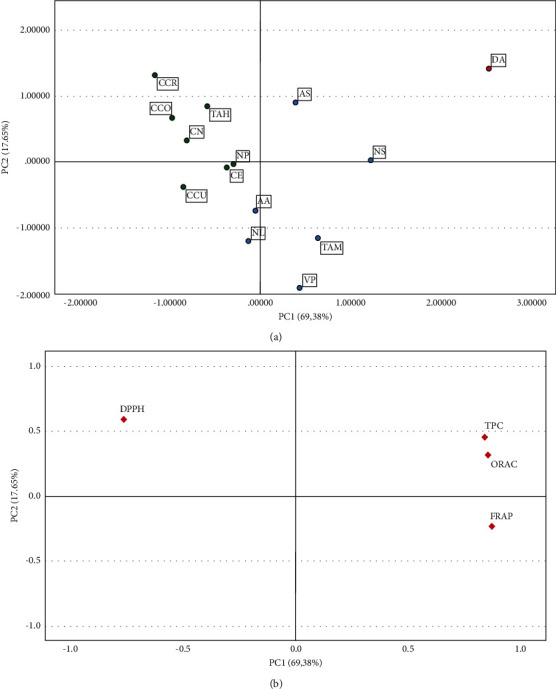
Reduction of multidimensional variables by principal component analysis (PCA) for fourteen different lichen species from cetrarioid clade. PCA allowed for the detection of similarities between samples and for establishing the main association between the variables. (a) PCA scores plot. (b) Loading plot. AA (*Allocetraria ambigua),* AS (*Asahinea scholanderi*), CCO (*Cetraria commixta*), CCR (*Cetraria crespoae*), CCU (*Cetraria cucullata*), CE (*Cetraria ericetorum*), CN (*Cetraria nivalis*), DA (*Dactylina arctica*), NL (*Nephromopsis laureri*), NP (*Nephromopsis pallescens*), NS (*Nephromopsis stracheyi*), TAH (*Tuckneraria ahtii*), TAM (*Tuckermannopsis americana*), and VP (*Vulpicida pinastri*).

Principal component analysis (PCA) and hierarchical cluster analysis (HCA) were determined to classify the fourteen lichen extracts based on its antioxidant properties (FRAP, ORAC, and DPPH assays) and phenolic content. PCA was applied to data based on a matrix correlation to eliminate the influence of the variance between variables with different units (i.e., DPPH and ORAC). Each parameter carried equal weight in principal component analysis. The PCA allowed for the detection of similarities between samples and for establishing the main association between the variables. The PCA results from Bartlett's test of sphericity indicate that variables are correlated with *p* < .001, and then our dataset was confirmed to be suitable for a data reduction technique. Principal component 1 (PC1) explained up to 69.38% of total variance, whereas principal component 2 (PC2) accounted for 17.65%, being 87.03% of the total variance (Figure 3(a)). Total variance explained table and scree plot were included in Supplementary Figure S1. The distance between lichen samples on the score plot explains the degree of differences and similarities in antioxidant activity and phenolic content. *Dactylina arctica*, *Asahinea scholanderi*, and *Nephromopsis stracheyi* were placed on right half up of the plot, *Tuckermannopsis americana* and *Vulpicida pinastri* were also at the right part, but half down of the plot, and finally, the other lichen species from cetrarioid clade were placed on right left half the plot, up (*Cetraria nivalis, Cetraria commixta, Cetraria crespoae, Tuckneraria ahtii*) and down (*Nephromopsis laureri, Allocetraria ambigua, Nephromopsis pallescens*, *and Cetraria cucullata*).  Figure 3(b) shows the relationship between the parameters studied. TPC, FRAP, and ORAC were clustered together, near each other, on the right side of the loading plot. Higher values of these variables indicate better total antioxidant activity. Oppositely, in the left hand of the loading plot at the very top of the figure, we find DPPH. This variable (expressed in IC_50_ values) inversely influences total antioxidant activity. Both plots allowed us to explain our results. The location of *D. arctica* diametrically opposite to the rest of the lichen samples was explained by the high levels of TPC, ORAC, FRAP *versus* low IC_50_ values of DPPH activity. Contrary to *D. arctica,* we observed *Cetraria cucullata, Cetraria nivalis, Cetraria commixta, Cetraria crespoae, Cetraria ericetorum, Tuckneraria ahtii,* and *Nephromopsis pallescens* which exhibited low total antioxidant capacity (ORAC, FRAP, DPPH) and low content of TPC. Only well-projected variables and individuals can be interpreted by PCA model. The parameter that measures the quality of the representation is cos^2^. High cos^2^ values indicate a good representation of the principal component. Conversely, low cos^2^ values show that variable and observations are not perfectly represented by the principal components [38]. Component's accountability expressed in percentages and cos^2^ values were included in Supplementary Tables S1 and S2. In our analysis, cos^2^ values for *A. scholanderi, C. ericetorum*, and *N. pallescens* were lower than 0.5 for both components and any assumptions drawn from this model related to these species have to be further investigated.

**Figure 4:**
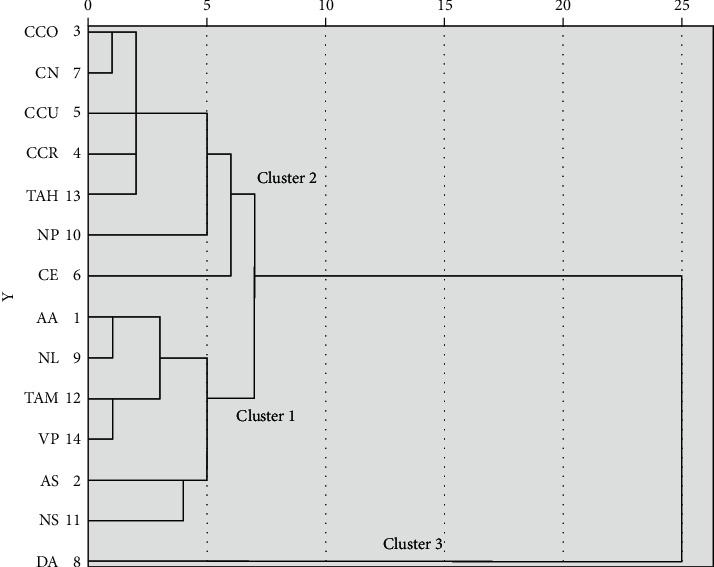
Dendrogram for lichen extracts from cetrarioid clade obtained from the hierarchical cluster analysis. Based on Euclidean distance, HCA examined similarities between lichen species and antioxidant activity. Samples are grouped in clusters in terms of their nearness or similarity. AA (*Allocetraria ambigua),* AS (*Asahinea scholanderi*), CCO (*Cetraria commixta*), CCR (*Cetraria crespoae*), CCU (*Cetraria cucullata*), CE (*Cetraria ericetorum*), CN (*Cetraria nivalis*), DA (*Dactylina arctica*), NL (*Nephromopsis laureri*), NP (*Nephromopsis pallescens*), NS (*Nephromopsis stracheyi*), TAH (*Tuckneraria ahtii*), TAM (*Tuckermannopsis americana*), and VP (*Vulpicida pinastri*).

The hierarchical cluster analysis (HCA), based on Euclidean distance, was used to examine similarities between lichen species and antioxidant activity. Samples are grouped in clusters in terms of their nearness or similarity. Dendrogram is shown in Figure 4 and the mean values of antioxidant activity and total phenol content of clusters of the HCA are in Table 3. Lichen species from cetrarioid clade were grouped into three clusters which confirm the PCA results. Cluster 1 included the lichen species *Nephromopsis stracheyi, Asahinea scholanderi, Tuckermannopsis americana, Vulpicida pinastri, Allocetraria ambigua, and Nephromopsis laureri.* This cluster was subdivided into two subclusters 1A and 1B. Subcluster 1A was constituted by *Nephromopsis stracheyi* and *Asahinea scholanderi*, which have moderate phenolic content and antioxidant capacity as shown in Table 3. Species from subcluster 1B presented low values of TPC but better values of DPPH suggesting the contribution of other compounds to its antioxidant capacity. On the other hand, seven lichens species (*Cetraria commixta*, *Cetraria nivalis*, *Cetraria cucullata*, *Cetraria crespoae, Cetraria ericetorum, Tuckneraria ahtii*, and *Nephromopsis pallescens*) composed cluster 2. The cluster 2 included lichen species, mostly consisted of Cetraria species, characterized by the lowest antioxidant properties and the lowest phenolic content values [31, 39, 40]. Cluster 3 is composed of a single species *Dactylina arctica*. The dendrogram showed that this species had a greater distance to the other clusters and therefore more differences. In phylogenetic studies, the species of *Dactylina* genus constitute their own subclade within cetrarioid clade [41].

Lichen extracts from cetrarioid clade were also screened for enzyme inhibitory activities [AChE and BuChE]. The IC_50_ values for each enzyme are shown in Table 4. The highest inhibitory activity of AChE was found for *Asahinea scholanderi* (IC_50_ = 0.11 mg/mL), *Tuckneraria ahtii* (IC_50_ = 0.15 mg/mL), and *Cetraria nivalis* (IC_50_ = 0.16 mg/mL), whereas the methanol extracts of *Cetraria commixta* and *Nephromopsis stracheyi* were less active (IC_50_ = 0.35 mg/mL). On the other hand, the lichens *Asahinea scholanderi* (IC_50_ = 0.29 mg/mL), *Cetraria cucullata* (IC_50_ = 0.31 mg/mL), and *Dactylina arctica* (IC_50_ = 0.42 mg/mL) were found to have the highest BuChE inhibitory activity. However, *Cetraria crespoae* (IC_50_ = 1.26 mg/mL), *Vulpicida pinastri* (IC_50_ = 0.89 mg/mL), and *Nephromopsis pallescens* (IC_50_ = 0.79 mg/mL) presented the lowest inhibitory activity of BuChE. The enzyme inhibitory activity for all methanol lichen extracts was high for AChE than for BuChE.

Previous studies showed AChE and BuChE inhibition potential of different lichen extracts. Hence, *Hypotrachyna formosana* inhibited AChE (31.8% for 25 *μ*g/mL, 39.5% for 50 *μ*g/mL, and 45.7% for 100 *μ*g/mL) and with less potency the enzyme BuChE (24.4% for 25 *μ*g/mL, 35.2% for 50 *μ*g/mL, and 41.2% for 100 *μ*g/mL) [31]. Also, Lee et al. reported AChE inhibition properties for *Umbilicaria esculenta* (22.4% for 1 mg/ml) [42]. However, although previous works have investigated AChE and BuChE inhibitory activity, research is very recent and limited.

**Figure 5:**
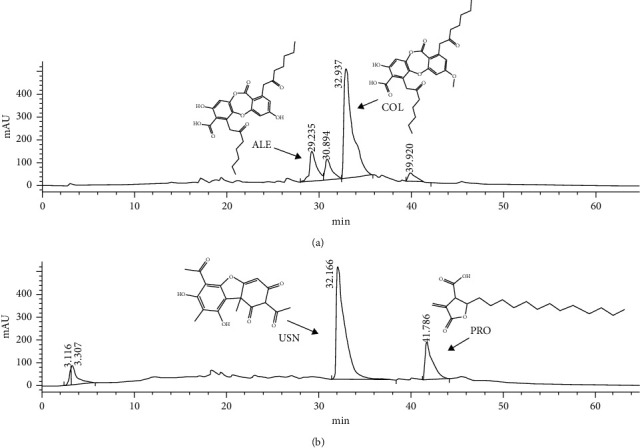
Representative HPLC-UV chromatogram (*λ* = 254 nm). Injected samples: lichen methanol extracts at 250 *µ*g/ml using a gradient elution with a 0.6 ml/min flow rate of 1% orthophosphoric acid in milli-Q water (a) and supergradient HPLC-methanol (b). (a) *Asahinea scholanderi* and (b) *Cetraria cucullata*. Retention times (TR) for each compound: ALE (29.24 min), COL (32.94 min), USN (32.17 min), and PRO (41.79 min). ALE (alectoronic acid), COL (*α*-collatolic acid), PRO (protolichesterinic acid), and USN (usnic acid).

The most active species on enzyme inhibition were *Cetraria cucullata* and *Asahinea scholanderi*. Identifying their secondary metabolites was carried out through HPLC analysis. Chemical composition analysis revealed as major secondary metabolites alectoronic acid (ALE) and *α*-collatolic acid (COL) in *A. scholanderi* (Figure 5(a)), and usnic acid (USN) and protolichesterinic acid (PRO) in *C. cucullata* (Figure 5(b)). Main peaks were identified by comparing their retention times with pure compounds (and lichen extracts with known composition) used as standards. Retention times, *λ* maximum spectra, and molecular formula are also included in Table 5.

**Figure 6:**
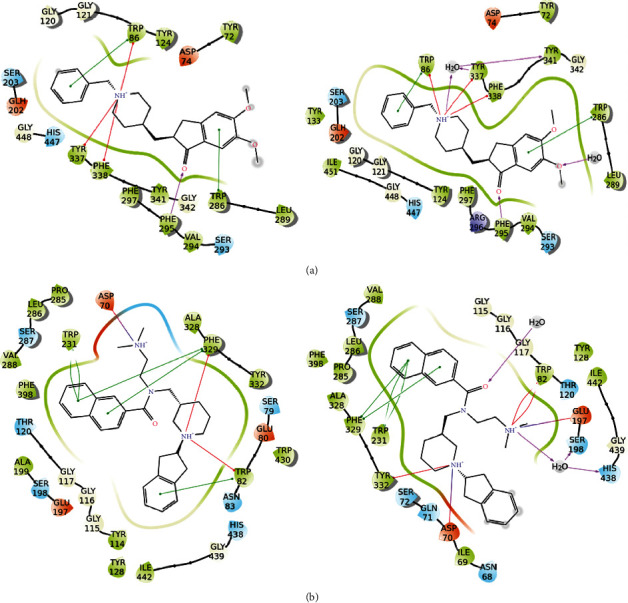
Protein-ligand interactions of the complexes formed by (a) AChE and donepezil and (b) BuChE and N-((1-2,3-dihydro-1H-inden-2-yl) piperidin-3-yl) methyl)-N-(2-(dimethylamino) ethyl)-2-naphthamide with (right panels) and without water molecules (left panels) inside the binding pocket. Hydrogen bonds are represented by pink arrows, *π*-*π* stackings are represented by green lines, red lines are *π*-cation interactions, and red-to-blue lines are salt bridges. Hydrophobic residues are in green, polar residues are in cyan, negatively charged residues are in red, positively charged ones are in blue, and glycine residues and water molecules are in white.

To better understand how the major secondary metabolites identified in *Asahinea scholanderi* (alectoronic acid and *α*-collatolic acid) and *Cetraria cucullata* (usnic acid and protolichesterinic acid) could inhibit AChE and BuChE enzymes, molecular docking studies were performed. Initially, Ach and the co-crystallized ligands were docked and re-docked against both AChE and BuChE. The binding was investigated with and without water molecules. In detail, the absolute binding affinity of Ach was higher for AChE (both hydrated and dehydrated) than for BuChE (Tables 6 and 7). The differences in pattern interaction with or without solvent molecules were evident in AChE, where Ach was better oriented in the hydrated binding site. In the case of co-crystallized molecules, both showed good re-docking results in terms of binding affinity and interactions with target (Tables 6 and 7, Figure 6). Moreover, the water network drove the compound in correct orientation in BuChE, creating a perfect overlapping between the docking pose and the co-crystallized ligand.

**Figure 7:**
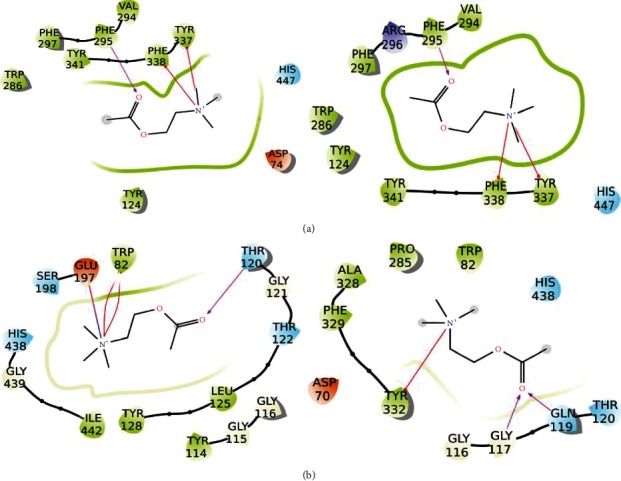
Protein-ligand interactions of acetylcholine with (a) AChE and (b) BuChE with (right panels) and without water molecules (left panels) inside the binding pocket. Hydrogen bonds are represented by pink arrows, red lines are *π*-cation interactions, and red-to-blue lines are salt bridges. Hydrophobic residues are in green, polar residues are in cyan, negatively charged residues are in red, positively charged ones are in blue, and glycine residues are in white.

**Figure 8:**
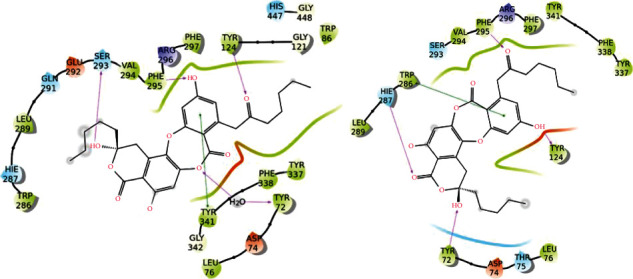
Protein-ligand interactions for alectoronic acid with AChE with (left) and without (right) water molecules included in the binding site. Hydrogen bonds are represented by pink arrows, and *π*-*π* stackings are represented by green lines. Hydrophobic residues are in green, polar residues are in cyan, negatively charged residues are in red, positively charged ones are in blue, and glycine residues and water molecules are in white.

For AChE, the key interactions inside the binding pocket are Trp86, Trp286, Tyr337, and Phe338 (pi-pi stacking); Tyr341 (hydrophobic); Tyr72, Ser293, and Phe295 (H-bond), and Ser203 and His447 (catalytic site). Among secondary metabolites, the highest affinity for AChE was found for alectoronic acid in the presence and absence of water molecules. This affinity and interaction network were similar and even superior to that of Ach (Figures 7 and 8; Table 6). However, donepezil which is clinically indicated for the treatment of Alzheimer's disease is more effective against AChE (docking score −18.5 kcal/mol with water and −15.7 kcal/mol without water) due to the presence of a positively charged nitrogen that promotes additional hydrogen bonds and *π*-cation interactions with the target (Figure 6(a)). Though results are not comparable to donepezil, alectoronic acid is a promising drug candidate to inhibit AChE. On the other hand, (2S,3R)-protolichesterinic acid is well inserted in the hydrophobic binding pocket; however, its moderate binding affinity and limited interactions with key amino acids discourage the hypothesis of a relevant binding to AChE (Figure S2).

**Figure 9:**
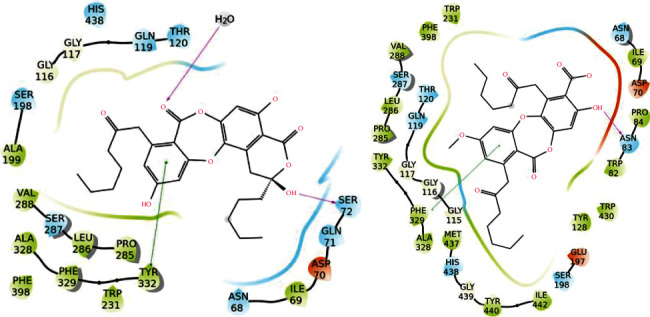
Protein-ligand interactions for alectoronic acid with BuChE with (left) and without (right) water molecules included in the binding site. Hydrogen bonds are represented by pink arrows, and *π*-*π* stackings are represented by green lines. Hydrophobic residues are in green, polar residues are in cyan, negatively charged residues are in red, positively charged ones are in blue, and glycine residues and water molecules are in white.

For BuChE, the binding cavity is delimited by Asn68, Asp74, Trp86, Gln119, Ser198, Trp231, Ala277, Leu286, Val288, Phe329, Ala328, and His438 [43]. The absolute binding affinities of the five secondary metabolites investigated against BuChE are lower compared to AChE with and without water. The binding affinities of these natural products showed a docking score higher or comparable to acetylcholine but lower than that of co-crystallized inhibitor N-((1-(2,3-dihydro-1H-inden-2-yl) piperidin-3-yl) methyl)-N-(2-(dimethylamino) ethyl)-2-naphthamide. Among lichen compounds, alectoronic acid showed the best results, even when compared to Ach (Table 7, Figures 7 and 9). In addition, (2S,3R)-protolichesterinic acid showed similar results to those obtained for acetylcholine, indicating a probable satisfying protein-ligand interaction (Figure S3).

Previous experimental studies have revealed that the dibenzofuran derivative usnic acid was a potential anticholinergic agent by inhibiting AChE (IC_50_ of 1.273 nM) and BuChE (IC_50_ of 0.239 nM) [44]. In addition, other secondary metabolites from different lichens to those of our study have shown to be promising cholinesterase inhibitors such as biruloquinone, isolated from *Cladonia mucilenta*, with inhibitory activity against AChE (IC_50_ of 27.1 *μ*g/ml) [45]. Also, lobaric acid, isolated from *Heterodermia* sp., inhibited AChE (IC_50_ of 26.86 *μ*M) and BuChE (IC_50_ of 36.76 *μ*M) [46]. Moreover, a new diacetate depsidone identified in *Lobaria pulmonaria* showed a moderate activity in AChE inhibition assays [47]. Indeed, docking studies with depsidones showed that depsidone scaffold could be used for the design of AChE inhibitors [45, 48].

Docking results can be related to *in vitro* enzyme inhibition assays. AChE inhibition values were better than BuChE, and moreover, docking scores for their compounds showed that less energy would be necessary for bonding with AChE. Indeed, *in vitro* assays showed that *A. scholanderi* has more activity than *C. cucullata* on both enzymes. Identified compounds of *A. scholanderi* (alectoronic acid and *α*-collatolic acid) showed better docking scores than *C. cucullata* ones (protolichesterinic acid and usnic acid).

## 4. Conclusions

Fourteen methanol extracts from lichens of cetrarioid clade were evaluated for its antioxidant capacity using multivariate statistical techniques and cholinesterase inhibitory activities combined with molecular docking. *Dactylina arctica* showed the highest total phenolic content and the highest ORAC value and FRAP value. Using PCA, 87.03% of the total variance was explained by two principal components. Moreover, HCA grouped lichen species into three clusters, highlighting the one that includes only the species *Dactylina arctica,* due to their antioxidant activity and total phenolic content, higher than the others.

On the other hand, *Asahinea scholanderi* and *Cetraria cucullata* extracts were the best inhibitors of AChE and BuChE. HPLC studies revealed that the major secondary metabolites of these lichen species were alectoronic acid and *α*-collatolic acid for *Asahinea scholanderi* and usnic acid and protolichesterinic acid for *Cetraria cucullata*. Molecular docking studies revealed that the compound alectoronic acid exhibited strong interactions with both AChE and BuChE with and without water molecules in the binding site. The compound (2S,3R)-protolichesterinic acid may also lead to good results as cholinesterase inhibitor because of the high lipophilicity of the binding cavity.

Our results concluded that *Dactylina arctica* stands out for its higher antioxidant capacities, followed by *Nephromopsis stracheyi, Tuckermannopsis americana, Vulpicida pinastri*, and *Asahinea scholanderi,* and the extracts *Asahinea scholanderi* and *Cetraria cucullata* act as AChE and BuChE inhibitors, being mandatory further investigation in cell culture and *in vivo* models to show their potential effectiveness in the treatment of neurodegenerative diseases.

## Figures and Tables

**Table 1:** Species of lichens from cetrarioid clade selected for this study (lichen name, origin, and MAF code).

Lichen species	Origin	MAF CODE
*Allocetraria ambigua* (C. Bab.) Kurok. and M.J. Lai	North Sikkim, India	22788
*Asahinea scholanderi* (Llano)	Russia	95154
*Cetraria commixta* (Nyl.) Th. Fr	Serra da Estrela, Beira Alta, Portugal	21548
*Cetraria crespoae* (Barreno and Vazquez) Karnefelt	Las Batuecas, Cáceres, Spain	10172
*Cetraria cucullata* (Bell.) Ach.	Krefelder Hütte, Austria	11754
*Cetraria ericetorum.* Opiz	Krefelder Hütte, Austria	11748
*Cetraria nivalis* (L.) Ach	Krefelder Hütte, Austria	11752
*Dactylina arctica* (Hook) Nyl.	North of Central Siberia, Krasnoyarsk Territory, Russia	96262
*Nephromopsis laureri* (Kremp.) Kurok.	Southern Siberia, Russia	22787
*Nephromopsis pallescens* (Schaer.) Y.S. Park	Himachal Pradesh, Dehla, India	22786
*Nephromopsis stracheyi* (C. Bab.) Müll. Arg.	North Sikkim, India	22785
*Tuckermannopsis americana* (Spreng) Hale	Maine, USA	19828
*Tuckneraria ahtii* Randlane and Saag	North Sikkim, India	22789
*Vulpicida pinastri* (Scop.) J.E. Mattsson and M.J. Lai	Alto del Peñón, Zamora, Spain	22790

**Table 2:** Yields, antioxidant activity (DPPH, ORAC, and FRAP), total phenolic content, and EAP index of methanol lichen extracts belonging to cetrarioid clade.

Lichen species	Yields (% w/w)	DPPH IC50 (*μ*g/mL)	ORAC value (*μ*mol TE/mg dry extract)	FRAP (*μ*mol of Fe^2+^ eq/g sample)	Total phenolic content (*μ*g GA/mg)	EAP Index	Rank
*Allocetraria ambigua* (C. Bab.) Kurok. and M.J. Lai	7.8 ± 3.6	895.9 ± 96.5^h,i,k,l,n^	1.3 ± 0.2^d,f,j^	19.9 ± 2.6^c,d,e,f,g,j^	52.4 ± 6.2	42.1	7
*Asahinea scholanderi* (Llano)	10.3 ± 1.3	1069.25 ± 45.8^h,i,k,l,n^	2.8 ± 0.1^a,c,d,e,f,g,i,j,m,n^	16.8 ± 2.2^c,f,g,j^	83.1 ± 5.6^a,c,d,e,f,g,i,n^	50.1	5
*Cetraria commixta* (Nyl.) Th. Fr	10.3 ± 1.2	1657.9 ± 110.4^a,b,e,f,h,j,i,k,l,n^	1.5 ± 0.03^d,e,f,j,m^	8.4 ± 2.3	44.9 ± 1.9	26.4	13
*Cetraria crespoae* (Barreno and Vazquez) Karnefelt	6.2 ± 1.6	2293.7 ± 54.9^a,b,c,e,f,g,h,i,j,k,l,m,n^	0.6 ± 0.02	12.6 ± 0.4	49.3 ± 8.6	26.5	14
*Cetraria cucullata* (Bell.) Ach.	8.7 ± 0.8	1262.8 ± 33.7^a,f,h,i,j,k,l,n^	0.7 ± 0.1	11.7 ± 1.7	39.3 ± 1.5	27.3	12
*Cetraria ericetorum*. Opiz	6.2 ± 0.1	830.4 ± 60.8^h,l,n^	3.6 ± 0.3	7.3 ± 0.6	41.7 ± 1.2	38	8
*Cetraria nivalis* (L.) Ach	5.6 ± 1.6	1485.1 ± 96.1^a,b,f,h,i,j,k,l,n^	1.5 ± 0.1^d,e,f,j^	10.4 ± 1.7	44.7 ± 4.2	29.6	11
*Dactylina arctica* (Hook) Nyl.	11.3 ± 0.3	346.3 ± 7.9	8.2 ± 0.6^a,b,c,d,e,f,g,i,j,l,m,n^	29.6 ± 1.8^a,b,c,d,e,f,g,i,j,m^	113.5 ± 6.7^a,b,c,d,e,f,g,I,j,k,l,m,n^	93.5	1
*Nephromopsis laureri* (Kremp.) Kurok.	7.5 ± 2.7	593.5 ± 53.5^n^	1.5 ± 0.1^d,e,f,j,m^	16.6 ± 2.1^c,f,g^	45.1 ± 5.1	44.6	6
*Nephromopsis pallescens* (Schaer.) Y.S. Park	8.1 ± 2.3	896.95 ± 137.1^n^	0.4 ± 0.04	11.6 ± 0.1	69.9 ± 5.7^c,e,f,g,i,^	36.1	9
*Nephromopsis stracheyi* (C. Bab.) Müll. Arg.	9.2 ± 3.1	595.3 ± 64.3^n^	3.8 ± 0.4^a,b,c,d,e,f,g,i,j,l,m,n^	27.1 ± 1.3^a,b,c,d,e,f,g,i,j,m^	84.2 ± 6.5^a,c,d,e,f,g,I,,l,n^	66.8	2
*Tuckermannopsis americana* (Spreng) Hale	9.8 ± 0.7	445.9 ± 45.8	2.3 ± 0.2^a,c,d,e,f,g,i,j,m,n^	25.1 ± 1.9^b,c,d,e,f,g,i,j,m^	60 ± 8.1	59.6	4
*Tuckneraria ahtii* Randlane and Saag	7.8 ± 3.6	1693.2 ± 112.2^a,b,e,f,h,i,j,k,l,m,n^	0.8 ± 0.1	14.3 ± 1.2^f^	63 ± 6.2	32.3	10
*Vulpicida pinastri* (Scop.) J.E. Mattsson and M.J. Lai	9.1 ± 1.7	283.7 ± 31.7	1.5 ± 0.1^d,e,f,j,m^	25.4 ± 2.3^b,c,d,e,f,g,i,j,m^	48.9 ± 4.8	61.8	3

Statistical significance (*p* < 0.05) is presented in letter superscripts: (a) *versus Allocetraria ambigua*; (b) *versus Asahinea scholanderi*; (c) *versus Cetraria commixta*; (d) *versus Cetraria crespoae*; (e) *versus Cetraria cucullata*; (f) *versus Cetraria ericetorum*; (g) *versus Cetraria nivalis*; (h) *versus Dactylina arctica*; (i) *versus Nephromopsis laureri*; (j) *versus Nephromopsis pallescens*; (k) *versus Nephromopsis stracheyi*; (l) *versus Tuckermannopsis americana*; (m) *versus Tuckneraria ahtii*; and (n) *versus Vulpicida pinastri*.

**Table 3:** *In vitro* antioxidant activity and total phenolic content of clusters of lichen species obtained by hierarchical cluster analysis (HCA).

	Cluster 1A	Cluster 1B	Cluster 2	Cluster 3	*p*-value^*∗*^	*p*-value^*∗∗*^
DPPH (IC_50_ *µ*g/mL)	832.3 ± 335.4	554.8 ± 260.3	1445.7^a,b,d^ ± 506.6	346.3 ± 0.2	0.002	<0.001
ORAC (*µ*mol TE/mg dry extract)	3.3^d^ ± 0.7	1.7^a,d^ ± 0.44	1.3^a,d^ ± 1.1	8.2 ± 0.1	0.028	<0.001
FRAP (µmol of Fe^2+^ eq/g sample	22^d^ ± 7.3	21.8^d^ ± 4.3	10.9^a,d^ ± 2.4	29.6 ± 0.2	<0.001	<0.001
Total phenolic content (*µ*g GA/mg)	83.7^d^ ± 0.8	51.6^a,d^ ± 6.3	50.4^a,d^ ± 11.6	113.5 ± 0.2	<0.001	<0.001

Results were expressed as mean ± SD. ^*∗*^ Levene's F test for equality of variances. ^*∗∗*^One-way ANOVA test. Statistical significance (*p* < 0.05) is presented in superscripts letter: (a) *versus* cluster 1A; (b) *versus* cluster 1B; (c) *versus* cluster 2, and (d) *versus* cluster 3.

**Table 4:** Acetylcholinesterase inhibition (IC_50_ values) and butyrylcholinesterase inhibition (IC_50_ values) of methanol lichen extracts belonging to cetrarioid clade.

Lichen species	AChE IC_50_ (mg/mL ± SD)	BuChE IC_50_ (mg/mL ± SD)
*Allocetraria ambigua* (C. Bab.) Kurok. and M.J. Lai	0.18 ± 0.015^b^	0.56 ± 0.016^b,e,h,k^
*Asahinea scholanderi* (Llano)	0.11 ± 0.006	0.29 ± 0.004
*Cetraria commixta* (Nyl.) Th. Fr	0.35 ± 0.017^a,b,d,e,f,g,h,i,j,l,m,n^	0.49 ± 0.018^b,e,k^
*Cetraria crespoae* (Barreno and Vazquez) Karnefelt	0.24 ± 0.05^a,b,e,f,g,h,i,l,m,n^	1.26 ± 0.004^a,b,c,e,f,g,h,i,j,k,l,m,n^
*Cetraria cucullata* (Bell.) Ach.	0.18 ± 0.014^b^	0.31 ± 0.001^b,e,k^
*Cetraria ericetorum*. Opiz	0.19 ± 0.016^b^	0.52 ± 0.013^b,e,k^
*Cetraria nivalis* (L.) Ach	0.16 ± 0.013^b^	0.75 ± 0.018^a,b,c,e,f,h,i,k,l^
*Dactylina arctica* (Hook) Nyl.	0.22 ± 0.005^b,g,m^	0.42 ± 0.008^b,e,k^
*Nephromopsis laureri* (Kremp.) Kurok.	0.17 ± 0.017^b^	0.65 ± 0.007^b,c,e,f,h,k,l^
*Nephromopsis pallescens* (Schaer.) Y.S. Park	0.22 ± 0.009^b,g,l,m^	0.79 ± 0.025^a,b,c,e,f,h,i,k,l^
*Nephromopsis stracheyi* (C. Bab.) Müll. Arg.	0.35 ± 0.002^a,b,c,d,e,f,g,h,i,j,l,m,n^	0.51 ± 0.004^b,d,e,f,h,I,j^
*Tuckermannopsis americana* (Spreng) Hale	0.17 ± 0.009^b^	0.49 ± 0.025^b,e,k^
*Tuckneraria ahtii* Randlane and Saag	0.15 ± 0.003^b^	0.70 ± 0.006^a,b,c,e,f,h,k,l^
*Vulpicida pinastri* (Scop.) J.E. Mattsson and M.J. Lai	0.19 ± 0.003^b^	0.89 ± 0.018^a,b,c,e,f,g,h,i,k,l,m^

Statistical significance (*p* < 0.05) is presented in superscript letters: (a) *versus Allocetraria ambigua*; (b) *versus Asahinea scholanderi*; (c) *versus Cetraria commixta*; (d) *versus Cetraria crespoae*; (e) *versus Cetraria cucullata*; (f) *versus Cetraria ericetorum*; (g) *versus Cetraria nivalis*; (h) *versus Dactylina arctica*; (i) *versus Nephromopsis laureri*; (j) *versus Nephromopsis pallescens*; (k) *versus Nephromopsis stracheyi*; (l) *versus Tuckermannopsis americana*; (m) *versus Tuckneraria ahtii*; (n) *versus Vulpicida pinastri.*

**Table 5:** Retention time (*t*_*R*_, HPLC) and UV spectral data of secondary metabolites identified in methanol extracts of lichens *A. scholanderi* and *C. cucullata*.

Secondary metabolite	Molecular formula	*t*_*R*_ (min)	*λ*_max_ (nm)
*α*-Collatolic acid	C_29_H_34_O_9_	32.79 ± 0.2	216/256/316
Alectoronic acid	C_28_H_32_O_9_	29.1 ± 0.18	214/254/316
Usnic acid	C_18_H_16_O_7_	32.28 ± 0.16	232/282
Protolichesterinic acid	C_19_H_32_O_4_	41.48 ± 0.4	230

**Table 6:** Predicted binding affinity between the compounds and the AChE.

Compound	Docking score (kcal/mol)	
	With H_2_O	Without H_2_O
Acetylcholine	−8.5	−7.4
Donepezil	−18.5	−15.7
Alectoronic acid	−10.8	−11.1
Atranorin	−8.7	−8.8
(2S,3R)-Protolichesterinic acid	−8.4	−7.9
Alpha-collatolic acid	−7.2	−9.9
Usnic acid	−5.2	−6.9

**Table 7:** Predicted binding affinity between the compounds and the BuChE.

Compound	Docking score (kcal/mol)	
	With H_2_O	Without H_2_O
Acetylcholine	−4.1	−5.1
N-((1-(2,3-Dihydro-1h-inden-2-yl) piperidin-3-yl) methyl)-N-(2-(dimethylamino) ethyl)-2-naphthamide	−15.9	−10.5
Alectoronic acid	−6.2	−6.6
Atranorin	−6.2	−6.9
(2S,3R)-Protolichesterinic acid	−5.4	−5.7
Alpha-collatolic acid	−5.5	−6.7
Usnic acid	−4.3	−7.5

## Data Availability

The data used to support the findings of this study are included within the article.

## Abbreviations

Ach: Acetylcholine
ANOVA: One-way analysis of variance
AChE: Acetylcholinesterase
BuChE: Butyrylcholinesterase
DPPH: 1,1-Diphenyl-2-picrylhydrazyl
EAP: Extract antioxidant potency
FRAP: Ferric-reducing antioxidant power
GA: Gallic acid
HCA: Hierarchical cluster analysis
ORAC: Oxygen radical absorbance capacity
ROS: Reactive oxygen species
PCA: Principal component analysis
TPC: Total phenolic content.

## Supplementary Materials

Supplementary Materials Figure S1: Scree plot and % of variance of the principal components extracted. Figure S2: protein-ligand interactions for (2S, 3R)-protolichesterinic acid with AChE with (A) and without (B) water molecules included in the binding site. The only hydrogen bond is represented by a pink arrow. Hydrophobic residues are in green, polar residues are in cyan, negatively charged residues are in red, positively charged ones are in blue, and glycine residues are in white. Figure S3: protein-ligand interactions for (2S,3R)-protolichesterinic acid with BuChE with (A) and without (B) water molecules included in the binding site. The only hydrogen bond is represented by a pink arrow. Hydrophobic residues are in green, polar residues are in cyan, negatively charged residues are in red, and glycine residues and water molecules are in white. Table S1: component's accountability (%) and quality of representation of each variable by the PCA model. Table S2: quality of representation for observations by the PCA model.
