# Supplementary material for: Phytochemical Characterization and Pharmacological Properties of Lichen Extracts from Cetrarioid Clade by Multivariate Analysis and Molecular Docking
Source: Evid Based Complement Alternat Med. 2022 Jun 2;2022:5218248. doi: 10.1155/2022/5218248 (PMC9187481; doi:10.1155/2022/5218248)
Supplement: Supplementary Materials — Figure S1: Scree plot and % of variance of the principal components extracted. Figure S2: protein-ligand interactions for (2S, 3R)-protolichesterinic acid with AChE with (A) and without (B) water molecules included in the binding site. The only hydrogen bond is represented by a pink arrow. Hydrophobic residues are in green, polar residues are in cyan, negatively charged residues are in red, positively charged ones are in blue, and glycine residues are in white. Figure S3: protein-ligand interactions for (2S,3R)-protolichesterinic acid with BuChE with (A) and without (B) water molecules included in the binding site. The only hydrogen bond is represented by a pink arrow. Hydrophobic residues are in green, polar residues are in cyan, negatively charged residues are in red, and glycine residues and water molecules are in white. Table S1: component's accountability (%) and quality of representation of each variable by the PCA model. Table S2: quality of representation for observations by the PCA model. [file 5218248.f1.docx]

#
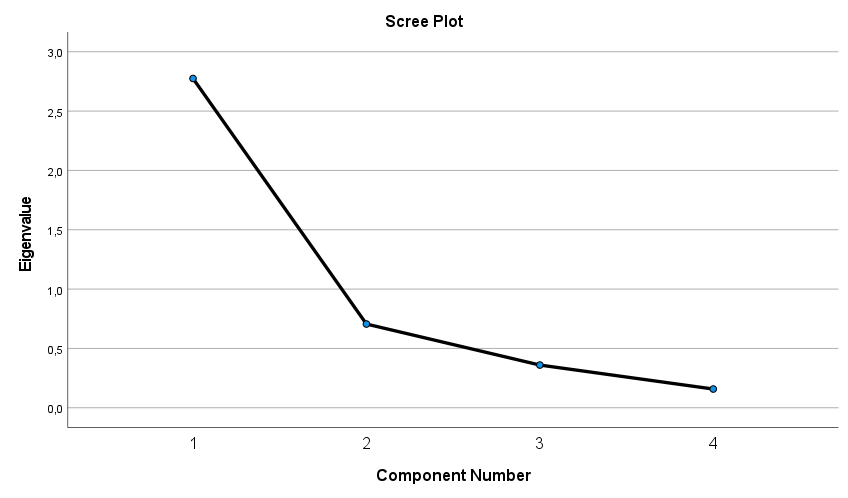
Supplementary material

| **Total variance explained** | | | | | | |
| --- | --- | --- | --- | --- | --- | --- |
| Component | Eigenvalues | | | Extracted Sums of Squared Loadings | | |
|  | Total | % of variance | Cumulative % | Total | % of variance | Cumulative % |
| 1 | 2.775 | 69.380 | 69.380 | 2.775 | 69.380 | 69.380 |
| 2 | .706 | 17.651 | 87.031 | .706 | 17.651 | 87.031 |
| 3 | .360 | 9.011 | 96.042 |  |  |  |
| 4 | .158 | 3.958 | 100.000 |  |  |  |
| Extraction method: Principal Component Analysis | | | | | | |

**Figure S1** Scree plot and % of variance of the principal components extracted**.**


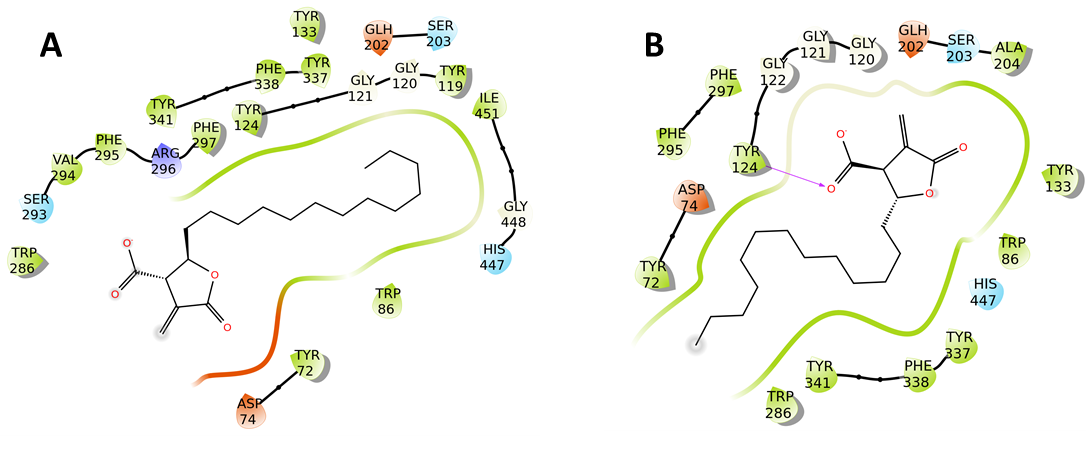


**Figure S2** Protein-ligand interactions for (2S, 3R)-protolichesterinic acid with AChE with (**A**) and without (**B**) water molecules included in the binding site. The only hydrogen bond is represented by a pink arrow. Hydrophobic residues are in green, polar residues are in cyan, negatively charged residues are in red, positively charged ones are in blue, and glycine residues are in white.


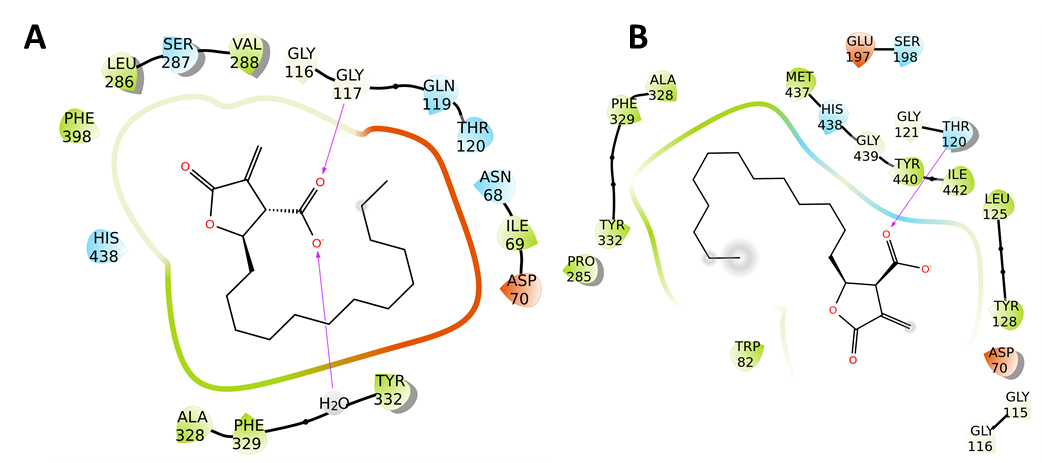


**Figure S3** Protein-ligand interactions for (2S,3R)-protolichesterinic acid with BuChE with (**A**) and without (**B**) water molecules included in the binding site. The only hydrogen bond is represented by a pink arrow. Hydrophobic residues are in green, polar residues are in cyan, negatively charged residues are in red, glycine residues and water molecules are in white.

|  | **Component´s accountability (%)** | | **Quality of representation(cos^2^)** | |
| --- | --- | --- | --- | --- |
| **Variables** | **PC1** | **PC2** | **PC1** | **PC2** |
| DPPH | 20.874 | 49.164 | 0.579 | 0.347 |
| ORAC | 26.265 | 14.178 | 0.729 | 0.100 |
| FRAP | 27.424 | 7.609 | 0.761 | 0.054 |
| TPC | 25.438 | 29.048 | 0.706 | 0.205 |

**Table S1.** Component´s accountability (%) and quality of representation of each variable by the PCA model.

**Table S2.** Quality of representation for observations by the PCA model.

|  | **Quality of representation(cos^2^)** | |
| --- | --- | --- |
| **Observations** | **PC1** | **PC2** |
| AA | 0.015 | **0.731** |
| AS | 0.342 | **0.448** |
| CCO | **0.907** | 0.038 |
| CCR | **0.647** | 0.210 |
| CCU | **0.940** | 0.048 |
| CE | **0.122** | 0.002 |
| CN | **0.851** | 0.102 |
| DA | **0.916** | 0.073 |
| NL | 0.040 | **0.885** |
| NP | **0.156** | 0.001 |
| NS | **0.951** | 0.000 |
| TAM | **0.511** | 0.429 |
| TAH | **0.508** | 0.269 |
| VP | 0.168 | **0.784** |
